# Radiologic exposomics: imaging the environmental imprint on cancer for precision oncology

**DOI:** 10.1007/s11547-026-02196-y

**Published:** 2026-03-05

**Authors:** Andrea Delli Pizzi, Massimo Caulo

**Affiliations:** 1 ITAB - Institute for Advanced Biomedical Technologies, “G. d’Annunzio” University, Chieti, Italy; 2 Department of Innovative Technologies in Medicine & Dentistry, “G. d’Annunzio” University, Chieti, Italy; 3 Unit of Radiology, “Santissima Annunziata” Hospital, Chieti, Italy; 4 Department of Neuroscience, Imaging and Clinical Sciences, “G. d’Annunzio” University, Chieti, Italy

**Keywords:** Radiologic exposomics, Radiomics, Environmental exposure, Air pollution, Cancer imaging, Precision oncology

## Abstract

Environmental exposures—such as airborne pollutants, metals, and urban stressors—contribute to cancer development and progression, yet their downstream biological effects remain difficult to characterize in vivo. Quantitative medical imaging may help fill this gap. Radiomics, in particular, offers access to tissue-level patterns shaped by chronic injury and microenvironmental remodeling. In this review, we discuss the rationale for linking geospatial exposure assessment with CT- and MRI-derived imaging biomarkers and outline how radiologic features may reflect processes associated with long-term environmental stress, including oxidative damage, inflammation, and metabolic or immune dysregulation. We also summarize epidemiologic evidence across major cancer types to contextualize where imaging–exposure integration is most plausible. A methodological workflow is presented, covering exposure assignment, imaging standardization, feature extraction, and strategies for harmonizing and modeling high-dimensional exposomic and radiomic data. Considerations related to confounding, data governance, and equity are also addressed, as these factors are integral to responsible implementation. Viewed in this light, imaging can be interpreted as an intermediate phenotype of the exposome—capturing aspects of tumor and peritumoral biology influenced by external stressors. This perspective may expand the role of radiology in precision oncology and generate new hypotheses about how environmental conditions shape cancer biology.

## Introduction

The concept of the exposome, first introduced by Wild in 2005, defines the totality of environmental, occupational, and lifestyle exposures an individual experiences over a lifetime [1]. Over the past two decades, exposomics has emerged as a complementary paradigm to genomics, aiming to unravel how environmental factors contribute to the development and progression of chronic diseases, including cancer [2, 3]. Integrating exposomics with genomics, metabolomics, and imaging represents the next frontier of systems oncology, where environmental and molecular determinants of cancer can be studied simultaneously. Traditionally, exposome research has relied on large-scale epidemiologic studies integrated with molecular “omics” (metabolomics, proteomics, epigenomics), but integration with imaging biomarkers remains largely unexplored [4].

In parallel, radiomics and quantitative imaging have transformed oncologic imaging in recent years. By extracting hundreds of mathematical features from CT, MRI, or PET scans, radiomics enables quantification of tumor shape, intensity, and texture patterns [5]. Numerous studies have demonstrated the predictive value of radiomics for therapy response, prognosis, and molecular characterization across cancer types such as lung, liver, prostate, and breast [6, 7]. Despite its success, radiomics has remained primarily confined to imaging itself, without systematic integration of environmental exposures that may shape the tumor phenotype.

The convergence of exposomics and radiology opens a new frontier. On the one hand, environmental pollutants—especially fine particulate matter (PM2.5), nitrogen dioxide (NO₂), and ozone (O₃)—have been associated with increased risk of lung, liver, pancreatic, and breast cancers [8, 9]. On the other, clinical imaging reflects not only intrinsic tumor properties but also the tumor microenvironment, which is influenced by chronic inflammation, hypoxia, and stromal remodeling—processes known to be modulated by environmental exposures [10]. In this sense, radiomic features, particularly those derived from peritumoral regions, could serve as intermediate phenotypic biomarkers of the exposome, translating environmental impact into quantifiable imaging patterns.

Furthermore, advances in satellite monitoring systems (Copernicus, Sentinel-5P, NASA MODIS) now allow estimation of individual exposure to air pollutants with kilometer-scale spatial resolution, enabling geospatial linkage with clinical and imaging data [11]. This technological convergence suggests a novel multimodal pipeline: linking historical environmental exposure for each patient with radiomic features of the tumor and its microenvironment, to improve prediction of oncologic outcomes such as treatment response, recurrence, and survival.

**Fig. 1:**
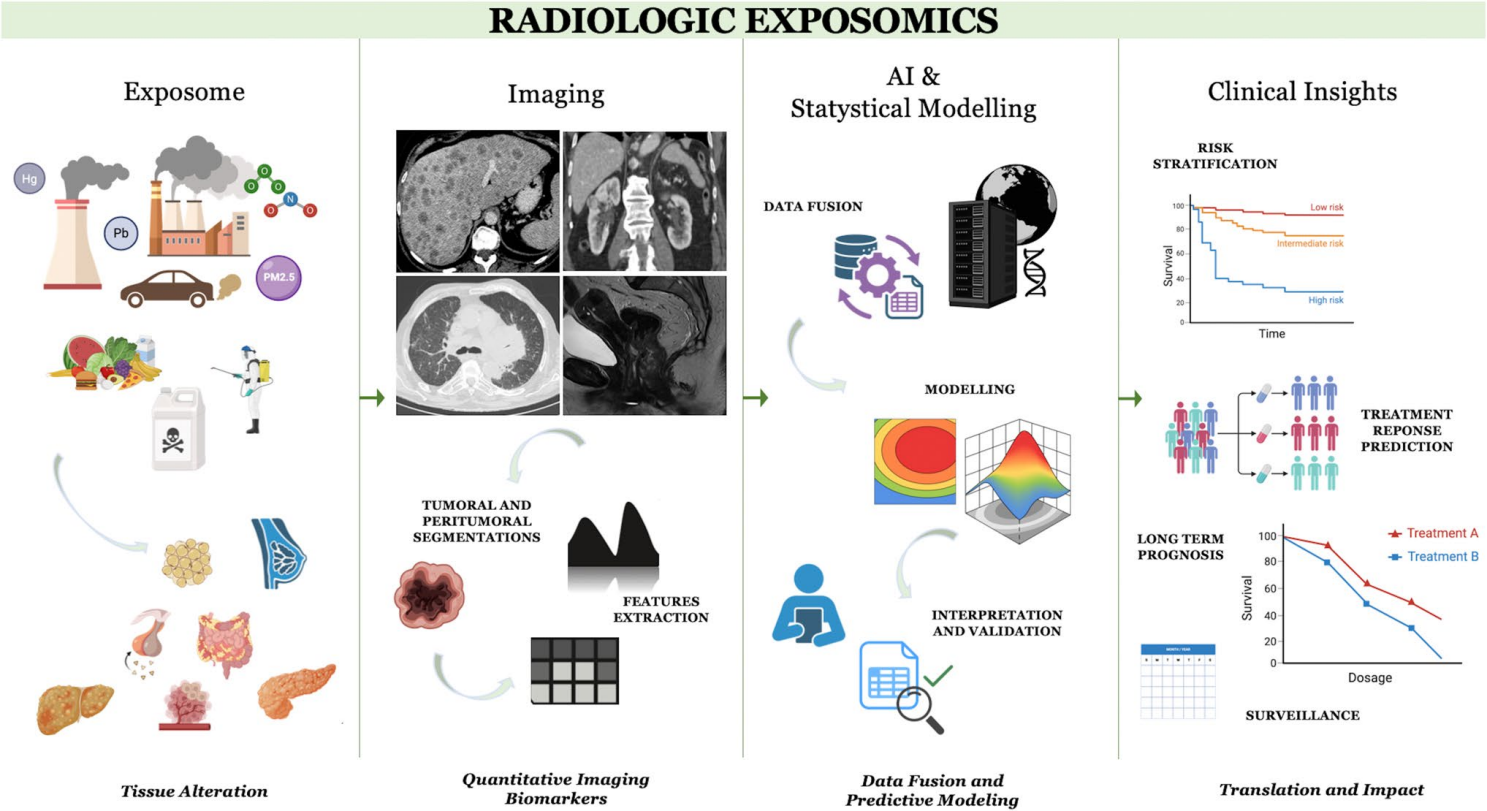
Conceptual Framework of Radiologic Exposomics. Radiologic exposomics integrates environmental exposure science with quantitative medical imaging to reveal how chronic external stressors shape tumor phenotype and clinical behavior. The framework illustrates a multiscale continuum: (1) exposome, encompassing air pollutants (PM_2.5_, NO_2_), heavy metals, volatile organics, and agro-industrial compounds, which induce oxidative, inflammatory, and metabolic tissue alterations; (2) imaging, where CT and MRI quantify these alterations through tumor and peritumoral segmentation and radiomic feature extraction; (3) AI & statistical modeling, which fuses exposomic and radiomic data via penalized regression and machine learning approaches to derive predictive, interpretable models; and (4) clinical insights, linking exposure-driven imaging phenotypes to risk stratification, therapeutic response, long-term prognosis, and surveillance. This integrative framework bridges population-level environmental epidemiology with patient-level precision oncology, highlighting how planetary exposures leave measurable imaging fingerprints in human disease

In this review, we will explore the conceptual foundations of the exposome, summarize current evidence linking environmental exposures and cancer risk, and highlight how radiomics can provide a unique phenotypic window into these processes. We illustrate this framework across several major cancer types—such as lung, liver, pancreas, cervix, rectum, and kidney—chosen for their clinical relevance, established imaging methodologies, and differing degrees of evidence linking environmental exposure to disease. Finally, we introduce radiologic exposomics, a new discipline that integrates quantitative imaging biomarkers with environmental exposure data. This framework aims to capture the downstream phenotypic imprint of the exposome, offering intermediate markers that reflect how environmental factors shape tumor biology and positioning imaging as a translational bridge between population-level exposome studies and precision oncology (Fig. 1).

## Exposome and oncology

The association between environmental exposures and cancer risk has been extensively documented in epidemiologic and public health literature. Ambient air pollution, particularly fine particulate matter (PM2.5), nitrogen dioxide (NO₂), and ozone (O₃), has been classified as a Group 1 carcinogen by the International Agency for Research on Cancer (IARC) [12]. While the strength of evidence varies across tumor sites, several cancers have emerged as being particularly susceptible to environmental pollutants. These heterogeneous susceptibilities suggest that distinct organs may serve as exposomic–radiomic paradigms, each reflecting specific pathways of phenotypic imprinting. In the next section, we explore these organ-level models in detail—linking external exposures to imaging phenotypes in lung, breast, liver, pancreas, cervix, rectum, and kidney.

### Lung cancer

Lung cancer represents the most robustly established link between environmental exposures and malignancy. Large cohort studies and meta-analyses have consistently demonstrated that long-term exposure to fine particulate matter (PM2.5) and nitrogen dioxide (NO₂) significantly increases the risk of lung adenocarcinoma, even among non-smokers [13–15]. These findings have been confirmed in extended analyses of the American Cancer Society cohort and in the multicenter ESCAPE project [14, 16]. The biological rationale involves oxidative stress, chronic inflammation, DNA damage, and immune dysregulation triggered by inhaled pollutants [17]. These processes promote genomic instability, epigenetic alterations, epithelial–mesenchymal transition, and impaired immune surveillance, ultimately facilitating malignant transformation.

Importantly, lung cancer has already been used as a “sentinel tumor” for environmental carcinogenesis, providing a model through which the complex interactions between exposure, host susceptibility, and tumor phenotype can be studied. From a radiologic perspective, the lung also represents an ideal testing ground for exposomic imaging, given the availability of standardized CT protocols, the direct interface with inhaled pollutants, and the extensive experience with radiomic characterization of pulmonary nodules and parenchymal alterations. Foundational studies have demonstrated that CT-based radiomics can capture microenvironmental heterogeneity and molecular correlates, making lung cancer a paradigmatic model for integrating environmental and imaging data within the exposome framework [6, 18].

### Breast cancer

Growing evidence indicates that air pollution also contributes to breast cancer risk, particularly for hormone receptor-positive (HR +) subtypes. Large-scale epidemiologic studies have shown that long-term exposure to NO2 and PM2.5 is associated with increased breast cancer incidence, even after adjusting for lifestyle and socioeconomic factors [13, 19]. Several mechanisms have been proposed, including endocrine disruption, oxidative stress, and local inflammatory responses, which may alter the mammary microenvironment and promote carcinogenesis. Of particular interest, population-based imaging studies have linked NO2 and PM2.5 exposure with increased mammographic density—an established imaging biomarker of risk [20]. This convergence of environmental, hormonal, and imaging factors makes breast cancer an ideal model for integrating exposome data with imaging-derived tissue phenotypes. Advanced breast MRI and contrast-enhanced mammography, both of which provide quantitative information on vascularity, permeability, and background parenchymal enhancement, may offer surrogate imaging markers of biological processes potentially modulated by environmental exposures [20–23]. These modalities have been shown to reflect microenvironmental factors such as hormonal activity, tissue perfusion, and neoangiogenesis.

### Liver cancer

The liver is a primary target for environmental exposures due to its central metabolic and detoxifying roles. Epidemiologic studies demonstrate associations between long-term exposure to fine particulate matter (PM2.5), volatile organic compounds, and industrial pollutants with increased incidence of hepatocellular carcinoma (HCC) [24]. Environmental toxicants have also been implicated in the development and progression of non-alcoholic fatty liver disease (NAFLD) and non-alcoholic steatohepatitis (NASH), which represent strong intermediaries between chronic exposure and hepatic malignancy [25]. Mechanistically, pollutants induce oxidative stress, mitochondrial dysfunction, and altered lipid metabolism, fostering a pro-carcinogenic microenvironment. This creates a compelling rationale for integrating exposome data with imaging-derived features of both liver parenchyma and tumors. Multiparametric MRI can capture fat, iron, fibrosis, and vascularization—parameters closely linked to metabolic and toxic stress. Radiomics studies have shown that quantitative features of HCC can predict microvascular invasion, recurrence, and survival [26–28]. The liver thus provides a model system where environmental and imaging biomarkers can be combined to trace the metabolic imprint of chronic exposure on tumor phenotype.

### Pancreatic cancer

Evidence connecting air pollution and pancreatic ductal adenocarcinoma (PDAC) is less extensive but growing. Recent population-based studies and meta-analyses have reported a significant association between chronic PM2.5 exposure and increased PDAC incidence [29]. The biological rationale is consistent with known mechanisms of oxidative stress, systemic inflammation, and metabolic dysregulation, which may promote tumor initiation and stromal activation. Given the highly desmoplastic and stromal nature of PDAC, it is biologically plausible that environmental exposures influence tumor–stroma interactions and the immune microenvironment, potentially generating measurable differences in imaging phenotypes. Radiomics studies have shown that peritumoral and stromal features on CT and MRI correlate with treatment response, fibrosis, and overall survival [30, 31]. These findings suggest that PDAC could serve as a model for exploring exposomic–radiomic relationships in desmoplastic tumors.

### Cervical cancer

Cervical cancer provides a unique model for radiologic exposomics. While persistent HPV infection remains the necessary cause of cervical carcinogenesis, progression and prognosis are significantly modulated by environmental and immunologic cofactors. Recent epidemiologic studies have linked ambient air pollution to HPV persistence and increased risk of cervical cancer [32, 33]. Mechanistically, exposure to fine particulate matter and traffic-related pollutants may promote oxidative stress, systemic inflammation, and impaired local immune response, thereby reducing viral clearance and enhancing susceptibility to malignant transformation. MRI, the gold standard for local staging, offers a robust platform for radiomic analysis of both intratumoral and peritumoral regions. The latter, in particular, may capture microenvironmental alterations influenced by systemic exposures, such as chronic inflammation or immune suppression. MRI-derived radiomics further captures stromal and microstructural characteristics that are relevant to treatment response and tumor–host interactions in cervical cancer [34, 35]. Although no direct evidence currently links environmental exposures to MRI-defined phenotypes, epidemiologic studies associating air pollution with HPV persistence and cervical dysplasia provide a plausible mechanistic rationale through which systemic exposures may influence the peritumoral microenvironment assessed by radiomics [32, 33]. Despite the widespread use of MRI in clinical practice, few studies have investigated how radiomic features might reflect environmental determinants in cervical cancer [34, 35]. This represents an untapped opportunity, where integration of exposure data with tumor and peritumoral imaging could reveal novel biomarkers for outcome prediction and risk stratification.

### Rectal cancer

Rectal cancer represents another promising setting for radiologic exposomics. Epidemiologic data indicate that environmental and lifestyle exposures—including air pollution, dietary habits, and sedentary behavior—may contribute to colorectal carcinogenesis through mechanisms of oxidative stress, dysbiosis, and chronic inflammation [36]. MRI is systematically used for local staging and for assessing treatment response, offering a robust framework for quantitative imaging analyses. Radiomics studies have shown potential in predicting outcomes, particularly pathologic complete response (pCR) after neoadjuvant chemoradiotherapy [37–39]. Importantly, characterization of the peritumoral compartment has emerged as a critical factor, capturing tumor–host interactions and microenvironmental remodeling that may reflect both intrinsic biology and external exposures. Building on these foundations, integrating exposome data (e.g., pollution metrics, diet, and lifestyle) with MRI-based tumor and peritumoral radiomics could offer new insights into how environmental factors shape tumor evolution and treatment response. Given the widespread adoption of standardized MRI protocols and the existence of large prospective cohorts, rectal cancer provides an ideal model for validating the concept of radiologic exposomics.

### Renal cancer

Renal cell carcinoma (RCC) provides an additional context for exploring radiologic exposomics. Epidemiologic studies have linked environmental exposures such as arsenic, cadmium, and fine particulate matter (PM2.5) with an increased risk of RCC, although the strength and consistency of evidence remain limited and heterogeneous [40–42]. Heavy metals and air pollutants may promote renal carcinogenesis through chronic oxidative stress, mitochondrial dysfunction, and inflammation within the renal parenchyma, particularly affecting tubular epithelial cells. From an imaging perspective, CT and MRI play a central role in RCC characterization, offering a fertile ground for radiomic investigation. Radiomics has demonstrated potential in differentiating histologic subtypes, predicting aggressiveness, and assessing treatment response [43–45]. However, the integration of exposomic data—such as long-term environmental pollutant exposure or occupational risk factors—into imaging-based models remains largely unexplored. This represents a critical opportunity for future research to elucidate how environmental toxicants may leave quantifiable phenotypic signatures detectable through quantitative imaging.

## Imaging oncology as a window on the exposome

Medical imaging has traditionally been regarded as a tool for diagnosis, staging, and treatment monitoring. However, in recent years, the concept of quantitative imaging and radiomics has shifted this paradigm by enabling systematic extraction of quantitative descriptors from clinical scans [46]. Radiomic features—encompassing intensity, shape, texture, and higher-order transformations—capture spatial heterogeneity and microstructural complexity that are often imperceptible to the human eye [47]. These features have been associated with biological processes such as cellular proliferation, necrosis, angiogenesis, and immune infiltration, suggesting that imaging can serve as a noninvasive surrogate for underlying tumor biology [48]. Radiomics applied to the tumor core has shown predictive and prognostic value across multiple cancers. For instance, studies in lung cancer demonstrated correlations between CT-derived texture features and EGFR or KRAS mutations [48]. In rectal cancer, MRI-based radiomics has been linked to response to neoadjuvant chemoradiotherapy [39]. These findings support the idea that imaging features can encapsulate the biological impact of both genetic and environmental determinants. However, focusing solely on the tumor neglects the crucial role of the surrounding tissue context. The peritumoral region represents a biologically rich compartment where tumor–host interactions unfold. Chronic inflammation, stromal remodeling, angiogenesis, and immune cell infiltration frequently extend beyond the tumor margins, and may be particularly sensitive to environmental exposures [49]. Peritumoral radiomics has already demonstrated added predictive value in breast and rectal cancer, where texture features extracted from a perilesional ring improved response prediction compared to tumor-only models [50, 51]. This supports the hypothesis that the peritumoral space acts as a “biological sensor” of external stressors, including those derived from the exposome. Environmental exposures exert their carcinogenic effects over time. Therefore, delta-radiomics, or the evaluation of longitudinal changes in imaging features, may provide a unique opportunity to capture dynamic tumor–environment interactions [50]. For example, monitoring changes in ADC-derived radiomic features during therapy could reflect not only treatment effect but also adaptive responses shaped by chronic environmental stress. Such temporal dynamics remain underexplored and represent a promising avenue for future exposomic imaging studies.

Another relevant concept is opportunistic imaging, whereby routine scans performed for unrelated indications are retrospectively mined for quantitative biomarkers [52]. Examples include estimating bone mineral density from abdominal CT, assessing muscle mass and sarcopenia from oncologic imaging, or quantifying hepatic fat from MRI acquired for staging [53]. Extending this approach to exposomics, clinical imaging archives could provide a wealth of phenotypic information reflecting environmental impact on organs and tissues, even in patients without cancer. Opportunistic imaging thus offers a scalable strategy to integrate exposome and imaging data in large retrospective cohorts. Taken together, these considerations suggest that imaging can be conceptualized as an intermediate phenotype of the exposome. Environmental exposures such as PM2.5, NO₂, and O₃ may not leave direct fingerprints in genomic sequences, but they do induce chronic biological alterations—fibrosis, inflammation, angiogenesis—that manifest as quantifiable patterns in medical imaging. By extracting and analyzing radiomic features from both tumor and peritumoral regions, researchers can potentially capture the downstream effects of environmental stressors at the tissue level. This novel integration positions radiology not merely as a diagnostic tool, but as a translational bridge linking environmental exposures to tumor behavior and clinical outcomes.

## Integration of environmental data and imaging

While the rationale for linking environmental exposures with imaging-derived tumor phenotypes is compelling, the methodological integration of these heterogeneous data streams represents a significant challenge. Environmental exposures are typically estimated at a population or geographic level, whereas imaging biomarkers are patient-specific and organ-focused. Bridging this gap requires careful attention to data sources, spatial and temporal resolution, and statistical harmonization.

Recent advances in satellite remote sensing and atmospheric modeling have dramatically improved the availability of high-resolution environmental data. Instruments such as Copernicus Sentinel-5P, MODIS (Moderate Resolution Imaging Spectroradiometer), and NASA’s OMI (Ozone Monitoring Instrument) provide daily global estimates of air pollutants including PM2.5, NO₂, and O₃ [54]. These satellite-derived estimates are increasingly combined with ground-based monitoring networks (e.g., EPA, EEA) and chemical transport models to generate robust, validated datasets of ambient air pollution exposure [55]. Other relevant sources include geographic information systems (GIS) capturing traffic density, industrial emissions, and green space, which have also been linked to cancer risk [56].

A major limitation of exposome studies has been the mismatch between the spatial resolution of exposure estimates and the individual-level nature of health outcomes. Satellite data now provide resolutions down to 1 km^2^ or finer, enabling more precise estimation of exposure at residential addresses [57]. However, patient mobility, occupational exposures, and indoor pollution remain sources of misclassification. Temporal resolution is equally important: Long-term averages (e.g., 3–5 years pre-diagnosis) are often most relevant for cancer, but short-term fluctuations may also influence acute biological responses [58].

The practical step involves geocoding residential history and matching it to environmental databases. Each patient can thus be assigned an individual estimate of cumulative exposure to pollutants during a relevant time window [59]. These exposure values can then be integrated with imaging features—tumor and peritumoral radiomics—into statistical or machine learning models [60]. Importantly, this approach allows testing whether environmental exposures are associated with specific imaging phenotypes (e.g., greater peritumoral heterogeneity in patients from high-PM2.5 regions) and whether combining both improves outcome prediction.

**Fig. 2:**
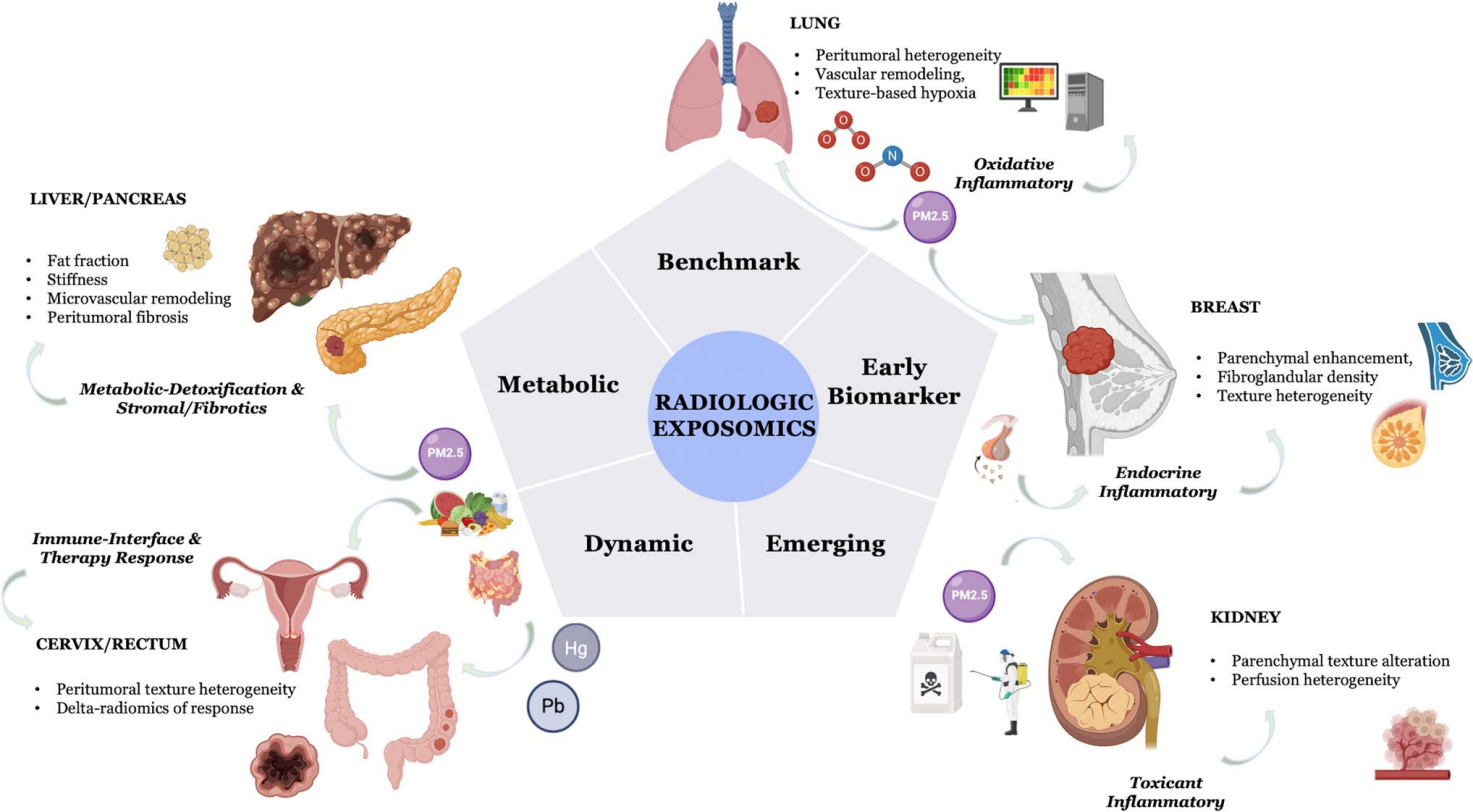
Organ-Based Paradigms in Radiologic Exposomics. Representative organ-based models illustrating how distinct exposomic paradigms manifest as imaging phenotypes. Benchmark model (Lung)—oxidative inflammatory paradigm: chronic PM₂.₅/NO₂ exposure leads to radiomic patterns of peritumoral heterogeneity and vascular remodeling. Early biomarker (Breast)—endocrine inflammatory paradigm: endocrine disruptors and air pollution modify breast density and parenchymal enhancement. Metabolic (liver/pancreas)—metabolic detoxification and stromal fibrotic paradigms: pollutants and dietary exposures drive steatosis, stiffness, and peritumoral fibrosis. Dynamic (cervix/rectum)—immune interface and therapy response paradigms: metals and chronic inflammation influence delta-radiomics signatures of treatment response. Emerging (Kidney)—Toxicant–inflammatory paradigm: heavy metal exposure produces parenchymal texture and perfusion changes on imaging. Together, these models illustrate the spectrum of environment–imaging interactions across cancer types, providing a framework for hypothesis-driven exposomic radiology

Integration of exposome and imaging data raises significant methodological challenges. First, both datasets are high-dimensional: Environmental data may include dozens of pollutants and land use indicators, while radiomics typically generates hundreds of features. This creates risks of multicollinearity and overfitting. Approaches such as penalized regression (LASSO, elastic net), principal component analysis, or feature selection based on reproducibility are recommended [61, 62]. Second, adjustment for potential confounders (smoking, socioeconomic status, comorbidities) is critical to isolate the true signal of environmental exposures [59]. Finally, validation in independent cohorts or through cross-validation techniques is essential to ensure generalizability (Fig. 2).

One of the most promising avenues lies in leveraging retrospective imaging archives from cancer centers and hospitals. These repositories often contain thousands of scans acquired for staging and follow-up, which can be reanalyzed opportunistically for radiomic features [63–65]. Coupled with publicly available environmental data, such archives offer a scalable opportunity to conduct exposomic imaging research without the need for prospective acquisition. This strategy could serve as a cost-effective entry point into this emerging field, while prospective studies are being designed [66].

## Proposed methodological pipeline

Developing an exposomic–radiomic framework requires a rigorous, multistage workflow ensuring spatial, temporal, and biological coherence between exposure and imaging data.

### Cohort definition and exposure assignment

Patient addresses or residential histories should be geocoded and linked to high-resolution exposure models derived from satellites, ground-based monitoring, or land use regression data. Combining multiple sources—such as atmospheric dispersion models, vegetation indices, and urban heat metrics—reduces spatial bias and improves coverage. Temporal granularity is equally important: long-term averages capture chronic exposure, whereas short-term fluctuations may relate to acute inflammatory responses. Ensemble or Bayesian kriging models can estimate exposure uncertainty at the individual level [67].

### Imaging data curation and segmentation

**Fig. 3:**
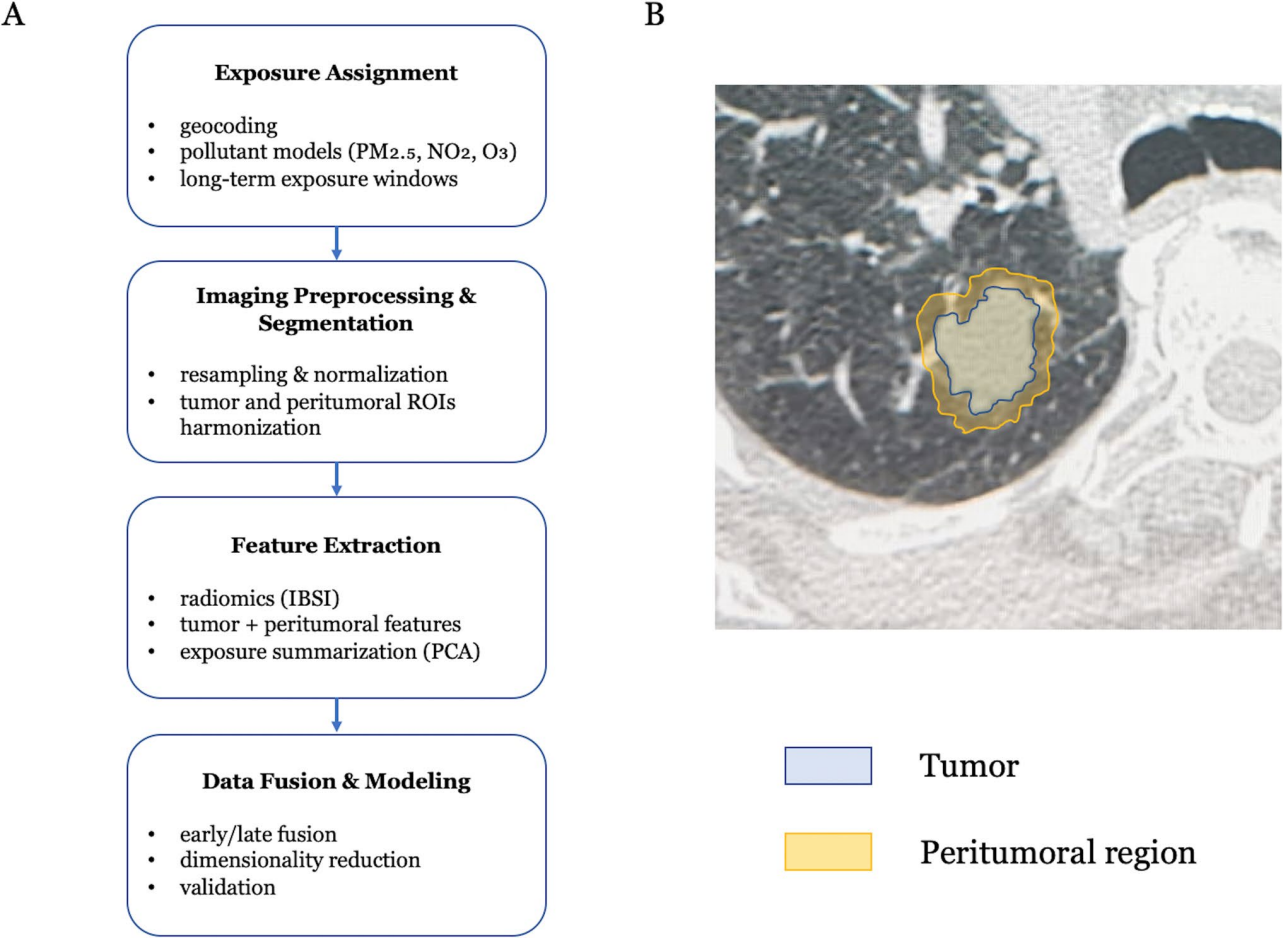
Conceptual framework for radiologic exposomics. (A) Overview of the radiologic exposomics pipeline. Individual-level exposure assignment is performed using geocoding, pollutant dispersion models (PM2.5, NO2, O3), and long-term exposure windows. Imaging data undergo preprocessing and harmonized segmentation of tumor and peritumoral regions. Radiomic features are then extracted according to IBSI standards from both tumor and peritumoral ROIs, while exposure variables are summarized using dimensionality reduction techniques (e.g., principal component analysis). Finally, imaging and exposure data are integrated through data fusion and modeling approaches, followed by validation. (B) Schematic illustration of tumor and peritumoral segmentation used for radiomic feature extraction. The intratumoral region (blue contour) represents tumor-intrinsic characteristics, whereas the peritumoral region (yellow contour) encompasses the surrounding microenvironment, which may reflect alterations influenced by systemic exposures

Imaging data must undergo standardized preprocessing, including voxel resampling, intensity normalization, bias field correction, and noise filtering. Both tumor and peritumoral compartments are segmented—manually, semiautomatically, or through deep learning algorithms—with attention to reproducibility and interobserver variability. The segmentation strategy, including the peritumoral ring, is illustrated in Fig. 3A. Cross-site harmonization (e.g., ComBat, GAN-based style transfer) mitigates scanner- or protocol-specific bias [68]. Multiparametric MRI or photon-counting CT provide complementary phenotypic dimensions.

### Feature extraction and data fusion

Radiomic features, extracted according to Image Biomarker Standardization Initiative (IBSI) recommendations, encompass intensity-based, morphological, textural, and wavelet descriptors [61]. Exposure features—covering chemical, physical, and contextual variables—can be aggregated into latent components using principal component analysis (PCA), exposure mixture modeling, or hierarchical clustering [2]. Early data fusion (concatenating exposure and radiomic matrices) and late fusion (combining model outputs) represent complementary integration strategies.

### Integration of exposure and imaging data

The integration of imaging-derived and exposure-related data relies on harmonizing two high-dimensional and partially correlated feature spaces. The overall exposomic–radiologic workflow, including exposure assignment, imaging preprocessing, feature extraction, and fusion strategies, is illustrated in Fig. 3B. Radiomic and exposomic matrices are first standardized and reduced through stability-based filtering and dimensionality reduction methods to retain reproducible and non-redundant descriptors [6, 61]. Exposure variables may be summarized using principal component analysis may be summarized using principal component analysis (a dimensionality reduction technique) or related approaches, while radiomic features undergo robustness-based selection. Depending on the analytical objective, integrated modeling may follow an early fusion strategy, in which reduced feature sets are concatenated, or a late fusion strategy, where modality-specific model outputs are combined. Overfitting is mitigated through penalized regression frameworks such as LASSO or elastic net and through cross-validated machine learning pipelines [69]. This step formalizes the integration of heterogeneous data streams prior to model development and validation.

### Modeling and validation

Multivariate regression and machine learning algorithms (LASSO, elastic net, random forests, gradient boosting, support vector machines) allow evaluation of incremental predictive value over imaging-only baselines [60]. Nested cross-validation and bootstrapping minimize overfitting. Calibration, decision curve, and reclassification metrics assess clinical utility. External validation across independent populations or federated multi-institutional networks is essential to ensure generalizability [70].

### Worked example scenarios

To demonstrate practical application of the proposed pipeline using currently available data resources, two representative study designs are outlined below.

*Lung Cancer Proof-of-Concept*—A retrospective cohort of lung cancer patients from large screening or staging datasets (e.g., NLST or PLCO) undergoes standardized CT radiomic analysis. Residential addresses are geocoded and linked to historical PM2.5 and NO2 exposure maps derived from satellite-based models. Tumor and peritumoral radiomic features are extracted and harmonized across scanners. Multivariable regression and machine learning models evaluate associations between long-term pollutant exposure and specific imaging phenotypes, and assess whether integrated exposomic–radiomic models improve prediction of survival or recurrence compared with radiomics alone. This design leverages existing imaging archives and publicly available exposure data.

*Rectal Cancer Clinical Translation Model*—Patients with locally advanced rectal cancer undergoing routine baseline and post-neoadjuvant MRI are retrospectively identified. Long-term environmental exposure (air pollution and urbanicity indices) is assigned through geocoded residential history. Tumor and peritumoral MRI radiomics and delta-radiomics are extracted. Integrated models assess whether exposomic variables add predictive value for pathologic complete response beyond standard MRI radiomics. This design relies on standard clinical imaging protocols and retrospective data resources.

### Confounding and statistical considerations

Environmental exposures covary with demographic, behavioral and socioeconomic factors that may induce systematic bias in exposure–imaging associations. To mitigate these effects, analytical models should incorporate adjustment strategies for socioeconomic status, smoking, comorbidities, and urbanicity, and use directed acyclic graphs to clarify causal structure and identify minimally sufficient adjustment sets [71]. Because environmental variables are often correlated, mixture modeling and penalized regression approaches may improve model stability and interpretability when handling multiexposure data [72].

### Causal and multiomic extensions

To move beyond association, causal inference frameworks—structural equation modeling, counterfactual analysis, directed acyclic graphs—can test mediation pathways linking exposure, imaging phenotype, and outcome. Integration with genomics, metabolomics, and immune profiling may elucidate the molecular correlates of radiologic exposomic signatures [73].

### Implementation and governance

Robust governance is required to ensure data privacy, security, and interoperability. Adherence to FAIR (Findable, Accessible, Interoperable, Reusable) principles and transparent documentation of preprocessing pipelines enable reproducibility across institutions [74]. Shared repositories, harmonized ontologies, and federated learning platforms could accelerate translation from proof-of-concept to clinical implementation [75].

#### Ethical, privacy, and equity considerations

The integration of geospatial exposure metrics with imaging and clinical data raises specific ethical and privacy considerations. Geolocation-linked health information may increase re-identification risk and therefore requires proportionate governance, data minimization, and controlled-access infrastructures [76]. Appropriate consent models depend on whether exposures are assigned at the individual or area level. Because environmental burdens disproportionately affect socioeconomically disadvantaged communities, exposomic imaging must also consider equity to avoid reinforcing structural disparities [77].

This modular workflow defines the methodological backbone of radiologic exposomics, uniting imaging science, environmental epidemiology, and data analytics within a reproducible and scalable framework—and laying the foundation for causal inference and translational precision oncology.

## Organ-based paradigms and representative models

Building on the conceptual framework outlined above, organ-specific case studies illustrate how distinct environmental exposures leave measurable phenotypic imprints in medical imaging. We propose five representative models of radiologic exposomics—benchmark, early biomarker, metabolic, dynamic, and emerging—each capturing a distinct biological paradigm through which environmental factors may shape imaging phenotypes (Fig. 2). These models are not mutually exclusive but complementary, collectively spanning the major axes of exposomic influence in oncology: metabolic detoxification, stromal fibrotic remodeling, immune interface modulation, dynamic treatment response, and toxicant-induced injury. Each model links a prototypical tumor type to its predominant exposure mechanism and characteristic imaging phenotype. Table 1 summarizes these paradigms, illustrating how environmental exposures manifest as quantifiable radiologic signatures across organ systems.

**Table 1:** Representative models in radiologic exposomics. This framework summarizes five representative models integrating environmental exposure data with oncologic imaging. Each model corresponds to a distinct biological paradigm—ranging from oxidative inflammatory injury to metabolic dysregulation, stromal remodeling, immune modulation, and toxicant-induced damage. The table outlines prototypical tumor types, dominant exposure mechanisms, and characteristic imaging phenotypes, highlighting how radiomics can capture the phenotypic imprint of chronic environmental stress across diverse organ systems

Model	Tumor Type(s)	Dominant Exposomic Paradigm	Main Environmental Drivers	Characteristic Imaging Phenotypes	Key Opportunities
Benchmark Model	Lung	Oxidative Inflammatory	PM_2.5_ NO_2_, O_3_	Peritumoral heterogeneity, vascular remodeling, texture-based hypoxia signatures	Validation of exposomic–radiomic pipelines in well-established datasets
Early Biomarker Model	Breast	Endocrine Inflammatory	Air pollution, endocrine disruptors	Parenchymal enhancement, fibroglandular density, texture heterogeneity	Integrating exposome metrics into screening and risk models
Metabolic Model	Liver, Pancreas	Metabolic Detoxification (liver)/Stromal Fibrotic (pancreas)	PM2.5, volatile organics, dietary/lipid exposures	Fat fraction, stiffness, microvascular remodeling, peritumoral fibrosis	Linking systemic metabolic stress and stromal remodeling via multiparametric MRI/CT
Dynamic Model	Cervix, Rectum	Immune Interface/Therapy Response	Air pollution, heavy metals, diet, chronic inflammation	Peritumoral texture heterogeneity, delta-radiomics of response	Prospective imaging of dynamic tumor–environment interactions
Emerging Model	Kidney	Toxicant–Inflammatory	Arsenic, cadmium, PM2.5, pesticides	Parenchymal texture alteration, perfusion heterogeneity	Discovery of new imaging biomarkers of toxicant-related carcinogenesis

The strength of the epidemiologic evidence linking environmental exposures to cancer risk varies substantially across organ systems. Table 2 summarizes the current level of epidemiologic, radiomic, and integrated exposome–imaging evidence across proposed tumor models and highlights which applications should be regarded as hypothesis-generating. While associations are well established for lung and breast cancer, the literature addressing pancreatic, renal, and cervical malignancies remains more limited and predominantly based on emerging observational and exposomic analyses. Accordingly, while lung, breast, liver, and rectal cancer models are supported by established epidemiologic and radiomic evidence, pancreas, cervical, and renal cancer models should currently be regarded as hypothesis-generating frameworks awaiting empirical exposome–imaging validation. Recent studies have reported associations between air pollution and pancreatic cancer [29, 78–80]. For renal cancer, multicohort and epidemiologic investigations have examined links with air pollutants, environmental toxicants, and heavy metals [41, 81–83]. Emerging evidence also suggests potential associations between ambient pollutants and cervical dysplasia or cancer [33, 84, 85]. Although these findings are preliminary, they support the biological plausibility for including these tumor types within a radiologic exposomics framework and highlight the need for dedicated imaging-based studies.

**Table 2:** Strength of evidence for exposome–imaging integration across tumor models. This table summarizes the current strength of evidence linking environmental exposures to cancer risk, radiomic imaging phenotypes, and their integrated application in radiologic exposomics. Pancreas, cervix, and kidney models should be regarded as hypothesis-generating frameworks

Tumor type	Epidemiologic link to exposure	Radiomic evidence	Existing exposome–imaging integration	Overall level of evidence
Lung	Strong (large cohorts, IARC)	Strong	Proof-of-concept feasible	Strong
Breast	Strong	Moderate	Early integration feasible	Moderate–Strong
Liver	Moderate	Moderate	Integration plausible	Moderate
Rectum	Moderate	Strong	Proof-of-concept feasible	Moderate–Strong
Pancreas	Emerging	Moderate	No direct integration yet	Emerging (hypothesis-generating)
Cervix	Emerging	Moderate	No direct integration yet	Emerging (hypothesis-generating)
Kidney	Emerging	Moderate	Exploratory	Speculative emerging (hypothesis-generating)

### Lung cancer: benchmark model

Representative of the oxidative inflammatory paradigm, lung cancer represents the benchmark model for radiologic exposomics. It is the most mature example of environmentally driven malignancy, supported by robust epidemiologic evidence linking long-term PM₂.₅ and NO₂ exposure to cancer risk, even in non-smokers [14]. This model exemplifies the oxidative and inflammatory pathways through which chronic air pollutants exert their carcinogenic and imaging-detectable effects. Beyond this causal foundation, lung cancer provides an unparalleled testing ground for large-scale integration of imaging and exposure data. Retrospective resources such as NLST and PLCO offer thousands of CT scans that can be geographically linked to regional pollutant maps, enabling evaluation of whether chronic exposure translates into measurable imaging phenotypes—such as peritumoral heterogeneity, vascular remodeling, or radiomic signatures of hypoxia [6]. These datasets also allow assessment of how integrating exposomic indicators with imaging biomarkers refines risk stratification and outcome prediction.

For these reasons, lung cancer serves as a methodological and biological benchmark, establishing analytic standards for exposomic–radiomic pipelines before their application to cancers with subtler environmental imprints. Its maturity and data availability make lung cancer the natural reference model for validating radiologic exposomics workflows across imaging centers.

### Breast cancer: early biomarker model

Representative of the endocrine inflammatory paradigm, breast cancer provides an exemplary model for radiologic exposomics focused on early risk phenotyping [13]. This paradigm illustrates how endocrine disruption and chronic low-grade inflammation can modify normal tissue composition long before tumor onset, creating measurable imaging phenotypes. Unlike most cancers, breast imaging is routinely performed in asymptomatic populations through large-scale screening programs, generating standardized longitudinal data at the population level. This infrastructure enables the integration of environmental exposure metrics—such as air pollution or endocrine-disrupting chemicals—with imaging-derived tissue biomarkers including breast density, parenchymal enhancement, and texture heterogeneity.

Radiomics applied to screening mammography or breast MRI has already shown that subtle quantitative features of normal tissue predict cancer risk and treatment response [22]. Integrating exposome data into these models could uncover how chronic environmental exposures shape tissue composition and imaging phenotype, offering a bridge between environmental epidemiology and imaging biomarkers of susceptibility. This early biomarker paradigm exemplifies how exposomic data could enhance screening personalization and prevention strategies.

As a result, breast cancer represents the early detection paradigm of radiologic exposomics, demonstrating how environmental signatures can be captured through routine imaging long before overt disease develops and how exposomic imaging may contribute to personalized prevention strategies.

### Liver and pancreatic cancer: metabolic model

Representative of the metabolic detoxification and stromal fibrotic paradigms, liver and pancreatic cancers together exemplify the metabolic axis of radiologic exposomics, where environmental stressors induce systemic biochemical and structural alterations that are phenotypically captured by imaging.

The liver, as the body’s primary metabolic filter, is directly exposed to xenobiotics, volatile organic compounds, and airborne particulates. Chronic exposure contributes to hepatocellular injury, steatosis, and fibrosis—conditions that precede or accompany hepatocellular carcinoma (HCC) [86, 87]. Multiparametric MRI provides a noninvasive window into these changes, quantifying fat, iron, perfusion, and stiffness. Radiomics of both tumor and background parenchyma could therefore quantify the cumulative metabolic burden imposed by environmental exposures, linking external pollutants to microvascular and parenchymal remodeling. This metabolic detoxification paradigm highlights how systemic exposomic injury can be measured through imaging biomarkers of tissue composition and perfusion.

The pancreas, in turn, represents the reactive and fibrotic component of this model. Pancreatic ductal adenocarcinoma (PDAC) develops within a highly desmoplastic and fibrotic stroma, shaped by chronic inflammatory and metabolic stress. Emerging evidence suggests that long-term PM₂.₅ exposure may increase PDAC incidence, reinforcing the concept that environmental stressors modulate tumor–stroma interactions. Radiomics applied to pancreatic CT and MRI has shown that peritumoral and stromal features predict survival and treatment response. Integrating exposomic metrics could clarify whether chronic exposure influences stromal architecture, potentially reflected in peritumoral heterogeneity.

Together, the liver and pancreas define the metabolic interface of radiologic exposomics, linking systemic environmental stress with tissue-specific imaging signatures of metabolic dysfunction and stromal remodeling. At present, this model should be considered hypothesis-generating, as no direct exposome–imaging studies have yet been performed in PDAC.

### Cervical and rectal cancer: dynamic model

Representative of the immune interface and therapy response paradigms, cervical and rectal cancers exemplify the dynamic dimension of radiologic exposomics—how environmental factors interact with host biology and treatment response over time. The cervix offers a unique model for investigating the immunologic interplay between environmental exposures and viral oncogenesis. Although cervical cancer is primarily driven by persistent HPV infection, chronic exposure to airborne pollutants and heavy metals has been linked to impaired immune surveillance, oxidative stress, and enhanced viral persistence. MRI, the reference standard for local staging, enables high-resolution assessment of both tumor and peritumoral compartments. Radiomics has already been applied to predict lymph node involvement and chemoradiotherapy response, and the integration of exposomic metrics could further elucidate how environmental stressors shape local immune and stromal microenvironments. Peritumoral features, in particular, may serve as imaging biomarkers of environmentally modulated immune imbalance, a hallmark of this immune interface paradigm.

The rectum, conversely, represents a clinically actionable setting for the therapy response paradigm. High-resolution MRI is routinely employed for baseline staging and post-treatment follow-up after neoadjuvant chemoradiotherapy. Radiomics has demonstrated that peritumoral texture features outperform tumor-only models in predicting pathologic complete response and long-term outcome. Because colorectal carcinogenesis and treatment sensitivity are influenced by chronic inflammation, diet, and pollutant exposure, combining exposomic and radiomic predictors could help explain interpatient variability in therapeutic response. Such integration is particularly relevant for organ-preserving “watch-and-wait” strategies, where imaging acts as both a treatment monitor and a sensor of ongoing environmental influence.

Together, cervical and rectal cancers define the dynamic paradigm of radiologic exposomics, in which longitudinal imaging captures the evolving interaction between environment, immunity, and therapy-induced tumor adaptation. Thus, the cervical cancer model currently represents a hypothesis-generating application of radiologic exposomics.

### Renal cancer: emerging model

Representative of the toxicant–inflammatory paradigm, renal cell carcinoma (RCC) represents an emerging frontier in radiologic exposomics, positioned at the intersection of environmental toxicology and quantitative imaging. Epidemiologic studies have suggested associations between RCC and chronic exposure to heavy metals—such as arsenic and cadmium—as well as fine particulate matter (PM₂.₅), although findings remain heterogeneous across populations [41]. Biologically, the kidney’s high perfusion rate and detoxification role make it particularly vulnerable to cumulative toxic insults, including oxidative stress, mitochondrial dysfunction, and DNA damage within tubular and interstitial compartments. From a radiologic perspective, CT and MRI are central to RCC detection and characterization, and radiomic approaches have demonstrated potential in predicting histologic subtype, molecular features, and clinical outcome. Integrating geospatial exposure data—such as heavy metal burden, water contamination indices, or air quality metrics—with radiomic descriptors of the tumor and adjacent renal parenchyma could uncover subtle imaging signatures of environmental injury. These may include alterations in parenchymal texture, perfusion heterogeneity, or changes in cortical–medullary differentiation reflecting chronic toxicant exposure. As an emerging model, RCC underscores the need for dedicated exposomic registries that integrate environmental monitoring with imaging phenotyping. This paradigm represents a crucial step toward identifying how chronic toxicant exposure leaves quantifiable imaging imprints within renal tissue, bridging environmental epidemiology and precision radiology. Accordingly, RCC presently remains an exploratory and hypothesis-generating model in radiologic exposomics.

## Challenges and limitations

Despite its promise, the integration of exposomics and radiomics faces several methodological, technical, and conceptual barriers that must be addressed before this approach can achieve clinical impact.

One of the most pressing limitations in radiomics is the lack of standardization in imaging acquisition, reconstruction, and feature extraction. Differences in MRI field strength, CT protocols, reconstruction kernels, and even contrast timing can significantly alter radiomic features. The Image Biomarker Standardization Initiative (IBSI) has provided guidelines to improve reproducibility, but adoption remains inconsistent [61]. For exposomic studies, this variability could obscure or confound subtle environmental effects. Prospective standardization and harmonization techniques (e.g., ComBat correction) are essential. Adherence to FAIR (Findable, Accessible, Interoperable, Reusable) data principles is essential to ensure reproducibility and interoperability across institutions [74].

Assigning individual exposure values from environmental datasets is inherently prone to misclassification. Satellite- and model-derived estimates typically provide area-level averages, while patients’ true exposures depend on indoor air quality, occupational environments, and personal mobility [55, 70]. Such misclassification tends to bias associations toward the null, potentially underestimating true effects. Prospective studies incorporating wearable sensors and personal monitoring may reduce this limitation, though feasibility is limited.

Both radiomics and exposomic data are high-dimensional and often intercorrelated. Environmental exposures are correlated with socioeconomic status, lifestyle, and comorbidities; radiomic features are correlated with acquisition parameters and tumor size. This creates risks of confounding and collinearity [61]. Multivariable models, penalized regression, and careful adjustment for known covariates are critical, but residual confounding cannot be excluded.

Many radiomics studies suffer from small sample sizes and lack of external validation [61]. This problem is amplified in exposomic imaging research, where both imaging and exposure data must be available. Without rigorous validation in independent cohorts, there is a risk of false positive associations. Multi-institutional collaborations and federated learning approaches may provide solutions.

Radiomics often generates hundreds of abstract features, many of which lack clear biological interpretation. Integrating these with exposome data risks creating “black box” models. For credibility, it is essential to emphasize interpretability by linking radiomic features to known biological processes (e.g., peritumoral heterogeneity with stromal inflammation) [61]. This translational anchoring will be crucial for acceptance by clinicians and public health researchers alike.

Exposome–imaging integration relies on geospatial linkage of patients’ residential addresses, raising concerns regarding privacy and data protection. Safeguards must ensure anonymization and secure handling of geolocation data. Ethical considerations also extend to equity, since environmental exposures are disproportionately higher in disadvantaged populations [70]. Exposomic imaging should therefore aim not only at scientific innovation but also at addressing health disparities.

### Role of radiologists in radiologic exposomics

Radiologic exposomics uses quantitative imaging as the principal phenotypic measure of tissue alterations associated with environmental exposures. Within this framework, radiologists have a central and non-substitutable role. They determine acquisition protocols required to ensure radiomic feature repeatability, supervise segmentation of tumor and peritumoral compartments, and oversee preprocessing and harmonization procedures needed to control scanner- and protocol-related variability. These steps are essential to guarantee robustness and biological interpretability of imaging biomarkers before integration with exposure data. Radiologists are further responsible for validation of automated segmentation and feature extraction pipelines, curation of imaging datasets, and verification of radiologic–pathologic consistency of derived imaging signatures. This expertise is required to avoid purely data-driven associations lacking clinical plausibility. In addition, radiologists retain responsibility for justification and optimization of examinations involving ionizing radiation, ensuring that exposomic imaging investigations comply with radiation protection principles and do not introduce unnecessary patient exposure.

Finally, radiologists provide the clinical interface through which exposure-informed imaging biomarkers can be translated into practice, including structured reporting, assessment of treatment response, and multidisciplinary tumor board decision-making. Radiologic exposomics therefore represents an extension of quantitative imaging research into environmental health applications, anchored in radiologic expertise.

### Future opportunities and perspectives

Integration of exposomic and radiomic data in oncology remains limited, but recent methodological and technological advances now enable systematic investigation of exposure–imaging relationships.

Future research should aim to combine exposomics, radiomics, and molecular omics (genomics, transcriptomics, proteomics, metabolomics). This multiomics framework would enable comprehensive characterization of how environmental exposures shape tumor biology at both molecular and phenotypic levels [70, 88]. For instance, environmental exposures could be linked to specific imaging phenotypes (e.g., peritumoral heterogeneity) and validated against molecular signatures of inflammation, hypoxia, or immune infiltration. Such integrative analyses may identify novel biomarkers that bridge epidemiology, biology, and imaging.

The high dimensionality of both radiomic and exposomic datasets makes them natural candidates for artificial intelligence (AI) and machine learning (ML) approaches. Multimodal deep learning models can integrate imaging features, environmental exposures, and clinical variables to improve prediction accuracy. Recent advances in explainable AI (XAI) offer tools to enhance interpretability, allowing clinicians to understand how environmental factors contribute to model predictions [70, 88]. This will be critical for clinical adoption.

Most exposomic studies rely on retrospective datasets. However, prospective longitudinal cohorts are needed to establish causality and temporal relationships. Imaging follow-up, combined with longitudinal exposure assessment, could reveal how chronic environmental stressors influence tumor evolution and treatment response over time. Such designs would also enable exploration of delta-radiomics as a biomarker of dynamic tumor–environment interactions [5].

If validated, exposomic imaging could enhance risk stratification and personalized oncology. For example, in rectal cancer, patients with high peritumoral heterogeneity and high PM2.5 exposure might be identified as poor candidates for organ-preserving strategies. In liver cancer, background parenchymal radiomics linked to pollutant exposure could refine surveillance strategies in at-risk populations [25, 39, 89]. By integrating environmental exposures into clinical decision-making, imaging could contribute to more equitable and tailored cancer care.

Beyond individual patients, exposomic imaging has implications for public health and planetary health. Linking environmental exposures to imaging biomarkers could help identify geographic “hot spots” where pollution leaves detectable biological imprints [9]. Such data could inform policy interventions, urban planning, and environmental regulations aimed at reducing cancer burden. The World Health Organization estimates that air pollution accounts for a significant fraction of global cancer mortality, underscoring the urgency of bridging radiology with environmental science [9].

To realize these opportunities, multidisciplinary collaborations will be essential. Radiologists, oncologists, epidemiologists, environmental scientists, and data scientists must work together to design robust exposomic imaging studies. Initiatives similar to The Cancer Imaging Archive (TCIA) could be extended to include environmental exposure metadata, fostering data sharing and reproducibility [70, 90]. Such networks will be crucial to move the field from proof-of-concept studies to large-scale impact. Radiologic exposomics thus unites the precision of clinical imaging with the scale of environmental health research, advancing a truly integrative vision of precision oncology.

### From research to clinical implementation

At present, radiologic exposomics remains predominantly a research activity requiring dedicated computational infrastructure, segmentation pipelines, exposure assignment platforms, and radiomic expertise that are not routinely embedded in standard clinical radiology workflows. Extraction, harmonization, and validation of radiomic features, even when supported by automated segmentation, remain resource-intensive tasks typically performed in research environments rather than during routine reporting. In the short to medium term, practical implementation is therefore expected within specialized imaging research units, where radiologists collaborate with environmental epidemiologists, atmospheric and exposure scientists, biostatisticians, and data scientists to construct integrated exposomic–radiomic datasets. Development of shared, multi-institutional imaging–exposure repositories and standardized preprocessing pipelines is a necessary prerequisite to derive reproducible imaging biomarkers and to define clinically meaningful and generalizable thresholds.

Translation of exposure-informed imaging biomarkers into clinical decision support or structured reporting will require prior external validation, cross-center protocol harmonization, and demonstration of biological interpretability. Within this translational pathway, radiologists remain central in supervising imaging quality, segmentation reliability, and clinical contextualization, ensuring that integration of environmental exposure information into oncologic imaging practice remains clinically justified.

## Conclusions

The integration of exposomics and radiomics represents a novel and largely unexplored frontier in oncologic research. Environmental exposures are recognized as major contributors to cancer risk and outcomes, yet their biological imprints have rarely been investigated through medical imaging. Radiomics, particularly when extended to peritumoral regions, provides a unique opportunity to capture the downstream effects of chronic exposures such as air pollution, translating them into quantifiable imaging phenotypes. Advances in satellite monitoring, geospatial modeling, and computational analytics now make it feasible to combine environmental data with imaging biomarkers at the individual patient level. While substantial challenges remain—including imaging standardization, exposure misclassification, confounding, and the need for external validation—the potential scientific impact is significant. Radiologic exposomics offers a framework to investigate how environmental stressors shape tumor phenotype, refine risk stratification, and improve understanding of variability in treatment response. Beyond individual patient care, this approach may also contribute to public health by identifying geographic areas where environmental exposures leave measurable biological imprints.

In summary, we propose radiologic exposomics as a distinct research framework leveraging imaging-derived phenotypes as intermediate biomarkers of environmental exposure. By systematically combining exposure assessment with tumor and peritumoral radiomics, this approach provides a reproducible methodological foundation to study how external stressors influence cancer biology. At present, radiologic exposomics remains primarily a research-oriented discipline, and translation into clinical practice will require standardized acquisition protocols, external validation of imaging biomarkers, and development of shared exposomic–radiomic datasets. Nonetheless, this paradigm establishes a conceptual and methodological bridge between environmental epidemiology and quantitative imaging, offering a realistic pathway toward integration of environmental determinants into precision oncology.

## Data Availability

No new data were generated or analyzed in this study. Data sharing is therefore not applicable.

## Abbreviations

ADC: Apparent Diffusion Coefficient
AI: Artificial Intelligence
CAD: Computer-Aided Diagnosis
CT: Computed Tomography
ComBat: Combining Batches (harmonization method for multicenter imaging data)
DCE-MRI: Dynamic Contrast-Enhanced Magnetic Resonance Imaging
DNA: Deoxyribonucleic Acid
EEA: European Environment Agency
EGFR: Epidermal Growth Factor Receptor
EPA: Environmental Protection Agency
ESCAPE: European Study of Cohorts for Air Pollution Effects
FAIR: Findable, Accessible, Interoperable, Reusable (data principles)
GIS: Geographic Information System
HCC: Hepatocellular Carcinoma
HER2: Human Epidermal Growth Factor Receptor 2
HPV: Human Papillomavirus
HR +: Hormone Receptor-Positive
IARC: International Agency for Research on Cancer
IBSI: Image Biomarker Standardization Initiative
ML: Machine Learning
MODIS: Moderate Resolution Imaging Spectroradiometer
MRI: Magnetic Resonance Imaging
NAFLD: Non-Alcoholic Fatty Liver Disease
NASH: Non-Alcoholic Steatohepatitis
NLST: National Lung Screening Trial
NO2: Nitrogen Dioxide
OMI: Ozone Monitoring Instrument
O3: Ozone
PCA: Principal Component Analysis
PDAC: Pancreatic Ductal Adenocarcinoma
PET: Positron Emission Tomography
PLCO: Prostate, Lung, Colorectal, and Ovarian Cancer Screening Trial
PM2.5: Fine Particulate Matter ≤ 2.5 μm
RCC: Renal Cell Carcinoma
RNA: Ribonucleic Acid
SVM: Support Vector Machine
TCIA: The Cancer Imaging Archive
WHO/ISUP: World Health Organization/International Society of Urological Pathology
XAI: Explainable Artificial Intelligence
pCR: Pathologic Complete Response
